# Eosinophilia in a Case of Strongyloides Stercoralis

**Published:** 1910-07-30

**Authors:** 


### EOSINOPHILIA IN A CASE OF STRONGYLOIDES STERCORALIS.

That certain intestinal parasites produce changes in the blood, possibly as the result of absorption of poisonous substances produced by the parasite in the intestines, is well known, and it is seen in marked degree in the severe cachexia that is produced by such parasites as the Anchylostomum duodenale and the Bothriocephalus latus; and amongst the most constant changes produced by the more serious parasites is eosinophilia. The value that the discovery of eosinophilia may be in diagnosis is obvious, and it will lead to confirmation of the diagnosis by examination of the fæces for the parasites themselves, or fragments, or for their ova. A patient suffering from these symptoms was recently under the care of Dr. Baetjer. She was a girl of twenty-two, whose main symptoms were shortness of breath, anæmia, and swelling of the ankles and feet, which had been coming on for eighteen months. There had been recurrent attacks of dyspnœa and weakness, with temporary improvement in the intervals, but at no time had there been diarrhœa or any intestinal symptoms. The only obvious thing about her was that her skin and mucous membranes were extremely pallid. Examination of her blood showed that the red corpuscles numbered 4,186,000 per cubic millimetre; the hæmoglobin amounted to 39 per cent. of the normal, the colour index being therefore 0.357; whilst the leucocytes numbered 11,400 per cubic millimetre, and the differential leucocyte count was as follows: Small lymphocytes and large lymphocytes together, 25 per cent.; polymorphonuclear cells, 30 per cent.; and eosinophiles, 45 per cent. So high a proportion of the coarsely granular eosinophilia corpuscles is very remarkable, and recalls some of the published statistics upon the subject in cases of trichinosis. The diagnosis in this case was confirmed by careful examination of the fæces by the sedimentation method with salt solution, both larvæ and ova being discovered.

In other cases of this affection there is nearly always eosinophilia, the percentage of eosinophiles varying from 6 to 13 per cent. in most patients, though sometimes reaching so high a figure as 38 per cent., or even 45 per cent. In several of the cases of strongyloid infection with eosinophilia it has been noted that these cells have decreased as the symptoms have abated and after the administration of anthelmintic drugs, the decrease corresponding with a rise of polymorphonuclear cells. A similar observation was made some years ago by Brown in cases of trichinosis, and it also occurred in cases of anchylostomiasis, such as those which have been reported happening amongst the miners in Cornwall and elsewhere.

